# Personality Type Influences Attentional Bias in Individuals with Chronic Back Pain

**DOI:** 10.1371/journal.pone.0147035

**Published:** 2016-01-20

**Authors:** Zoë C. Franklin, Paul S. Holmes, Nickolas C. Smith, Neil E. Fowler

**Affiliations:** Manchester Metropolitan University, Centre for Health, Exercise and Active Living, Crewe Campus, Crewe Green Road, Crewe, Cheshire, CW1 5DU, United Kingdom; University of Florida, UNITED STATES

## Abstract

Attentional biases reflect an individual’s selective attention to salient stimuli within their environment, for example an experience of back pain. Eysenck suggests that different personality types show different attentional biases to threatening information. This study is the first to test Eysenck’s theory within a chronic back pain population by investigating the attentional biases of four different personality types using a back pain specific dot-probe paradigm. Participants were 70 volunteers (45 female) recruited from a back rehabilitation program at an NHS Trust. The four groups were selected on their trait anxiety and defensiveness scores: defensive high-anxious; high-anxious; repressor and non-extreme. Participants completed a dot probe task comprising 20 practice trials and 250 experimental trials. The experimental trials contained 100 threat-neutral pairs, 100 positive-neutral pairs and 50 neutral-neutral image pairings. The threat images were taken from the Photograph Series of Daily Activities (PHODA) and the neutral and positive images from the International Affective Picture System (IAPS) image bank. The results provided partial support for Eysenck’s theory; defensive high-anxious individuals showed an attentional bias for threatening information compared to high-anxious individuals who demonstrated no bias. Repressors showed an avoidant bias to threatening images and an attentional bias to positive stimuli relative to neutral images. The clear difference in responses demonstrated by high-anxious individuals who vary in defensiveness highlight the need for separate investigation of these heterogeneous groups and help to explain the cognitive processes of defensive high-anxious individuals within a pain population. The demonstration of an attentional bias in this group to threatening information could explain why defensive high-anxious individuals are more likely to re-present for treatment.

## Introduction

Individual representations of pain are influenced by individual tendencies on how we process information. Biases relating to whether or not to attend to particular information, interpretation of it and memory define how each person responds to the experience of chronic pain. Theories of attention and pain predict that individuals with chronic pain will display attentional biases towards pain-related information [1]. An attentional bias can be considered as a selective attention towards or away from a stimulus, which is both specific and salient to the current environment. Attentional biases have been investigated in populations with anxiety disorders [2], depression [3], chronic headache [4] and other clinical groups [5,6] and they may have important therapeutic implications for the patient and therapist. Studies which have investigated the attentional biases of individuals with chronic pain have found an enhanced attention compared to controls, however, the effect sizes are relatively small (r = 0.1–0.3). Studies investigating the attentional biases of individuals with a chronic musculoskeletal condition have tended to test the population as a whole and not as differentiated groups based on variations in anxiety [4,6–10]. This may be important since individual differences in attentional bias underlie vulnerability to clinical anxiety and vigilance for pain-related symptoms [11].

Within chronic pain patients, anxiety has been shown to be important in the development of pain and the resulting disability. Eysenck [12] has theorized that the subjective experience of anxiety is influenced by four sources of information: (i) the cognitive appraisal of the situation; (ii) the negative cognitions that arise about possible, future events (e.g. worries); (iii) the individual’s interpretation of their own behavior; and (iv) the attention to and interpretation of the individual’s physiological activity. There are two main assumptions within this theory that serve to influence the processing of the four sources of information. First, individual differences in trait anxiety and defensiveness affect the operation of attentional and interpretive biases that serve to either magnify or minimize the processing of threat-related stimuli and second, the biases in cognition are affected by the prevailing level of state anxiety.

Weinberger, Schwartz and Davidson [13] suggested that low and high-trait anxious individuals can be split into four heterogeneous groups based on their defensiveness scores. Low trait anxiety combined with low defensiveness scores reflect truly low-anxious groups, whilst those with low trait anxiety but high defensiveness scores are defined as repressors. High trait anxious individuals can also be divided into two groups, high trait anxiety scores combined with low defensiveness scores reflect the truly high-anxious group, and those with a combination of high trait anxiety and high defensiveness are known as defensive high-anxious individuals. Eysenck [14] suggested that repressors have an avoidance bias to threat that results in avoidance of negative or threatening cues. In contrast, low-anxious individuals show no cognitive bias. Eysenck postulated that high-anxious individuals exhibit both attentional and interpretive biases that amplify potential threat and lead them to interpret ambiguous stimuli as threatening. Unfortunately, due to their relative scarcity in the general population, few studies have considered the responses of defensive high-anxious individuals separately from their high-anxious counterparts with the consequential assumption that they respond similarly to high-anxious individuals (Eysenck, 1997).

Notwithstanding the concerns for the lack of a defensive high-anxious group, there are several studies supporting Eysenck’s predictions regarding attentional biases in high-anxious, low-anxious and repressors within the general population [15–17]. There are, however, some theoretical inconsistencies in these studies, with some using words [18], whereas others have used faces [19]. The variation in the findings highlights the need for further research to consider the combination of anxiety and defensiveness when investigating the attentional biases of individuals and for the use of clinically relevant sample populations and stimuli.

Due to their relative scarcity in the general population (7–10%), defensive high-anxious individuals have often been either omitted from studies [20], or combined into a single group with high-anxious individuals [21]. In groups with chronic musculoskeletal pain conditions (back pain and chronic fatigue syndrome), however, the proportion of defensive high-anxious individuals has been shown to be much higher (39–46%) [22,23]. Franklin, Smith and Fowler [24] recently showed that defensive high-anxious individuals were more likely to utilize a variety of treatment options compared to the other three personality types. Suggesting that this group may be more persistent within the care system and thus more likely to be referred to chronic pain management groups. Therefore, it may be helpful to identify whether defensive high-anxious individuals also show an attentional bias towards pain-related threat. Automatic tendencies to attend to pain related information may cause the individual to be more susceptible to comorbid conditions such as depression and potentially adopt maladaptive coping strategies. A better understanding of the varied attentional biases of patients from different personality groups may help inform targeting of pain management strategies.

The present study aims to test Eysenck’s (1997) theory in a clinical setting by examining whether the attentional biases of defensive high-anxious individuals to pain related stimuli differ to the other personality groups in a population with chronic back pain.

## Materials and Methods

### Participants

Participants were 70 volunteers (45 female) recruited from a back rehabilitation program at an NHS Trust in the NW of England, UK and a control group of 20 asymptomatic individuals. Table 1 shows the demographic data for the patient and asymptomatic control groups. All participants from the back rehabilitation program reported suffering from back pain for more than three months. Ethical approval was provided by the National Research Ethics Service (NRES) Committee North West—Greater Manchester Central and by the Manchester Metropolitan University Sport and Exercise Science department ethics committee. All participants provided written informed consent.

**Table 1:** Summary of the mean (±SD) demographic data for the defensive high-anxious, high-anxious, repressor, non-extreme and asymptomatic individu als.

	Patients				Controls
	Defensive high-anxious (n = 18)	High-anxious (n = 11)	Repressor (n = 9)	Non-extreme (n = 29)	Asymptomatic controls (n = 20)
Age (years)	50.7 (± 12.9)	42.0 (± 15.5)	54.0 (± 18.0)	50.9 (± 12.4)	36.1 (± 10.5)
Sex	12 females 6 males	9 females 2 males	5 females 4 males	19 females 9 males	9 females 11 males
Defensiveness	8.5 (± 0.7)	3.5 (± 0.6)	8.5 (± 0.7)	6.6 (± 1.4)	5.5 (± 2.1)
Trait anxiety	50.3 (± 8.4)	52.2 (± 8.9)	26.2 (± 3.4)	38.5 (± 7.6)	36.6 (± 8.6)
State anxiety	31.7 (± 8.6)	29.5 (± 6.2)	22.7 (± 3.6)	30.6 (± 8.2)	27.3 (± 7.4)

#### Patients

Participants were split into personality groups based on their STAI and the MC-SDS scores. Based on previous research (Franklin et al., 2014) the groups were determined according to the following criteria: (i) defensive high-anxious (DHA; n = 18), trait anxiety scores 42 and above, and defensiveness 8 and above; (ii) high-anxious (HA; n = 11), trait anxiety scores 42 and above, and defensiveness 4 and below; (iii) repressors (REP; n = 9), trait anxiety scores 30 and below, and defensiveness 8 and above. The low-anxious group were excluded from analysis because only three individuals were identified (trait anxiety scores below 30 and defensiveness below 4). The non-extreme individuals (n = 29) were participants who scored in the mid-range for trait anxiety and defensiveness.

#### Asymptomatic controls

A control group of asymptomatic participants (n = 20) was recruited from contacts within the university and the local area and asked to perform the same tasks as the patient group. Participants were all individuals who were either low-anxious (trait anxiety scores below 30 and defensiveness below 4) or scored in the mid-range for anxiety (31–41) and defensiveness (5–7). As participants were free from any current or past history of back pain, it was anticipated that the PHODA images would hold no specific or relevant threatening content. The recruitment of asymptomatic control participants enabled a comparison with any biases seen in the patient groups indicating either selective attention to or avoidance of back-pain relevant threatening information.

### Measures

#### Dot probe paradigm

**Fig 1:**
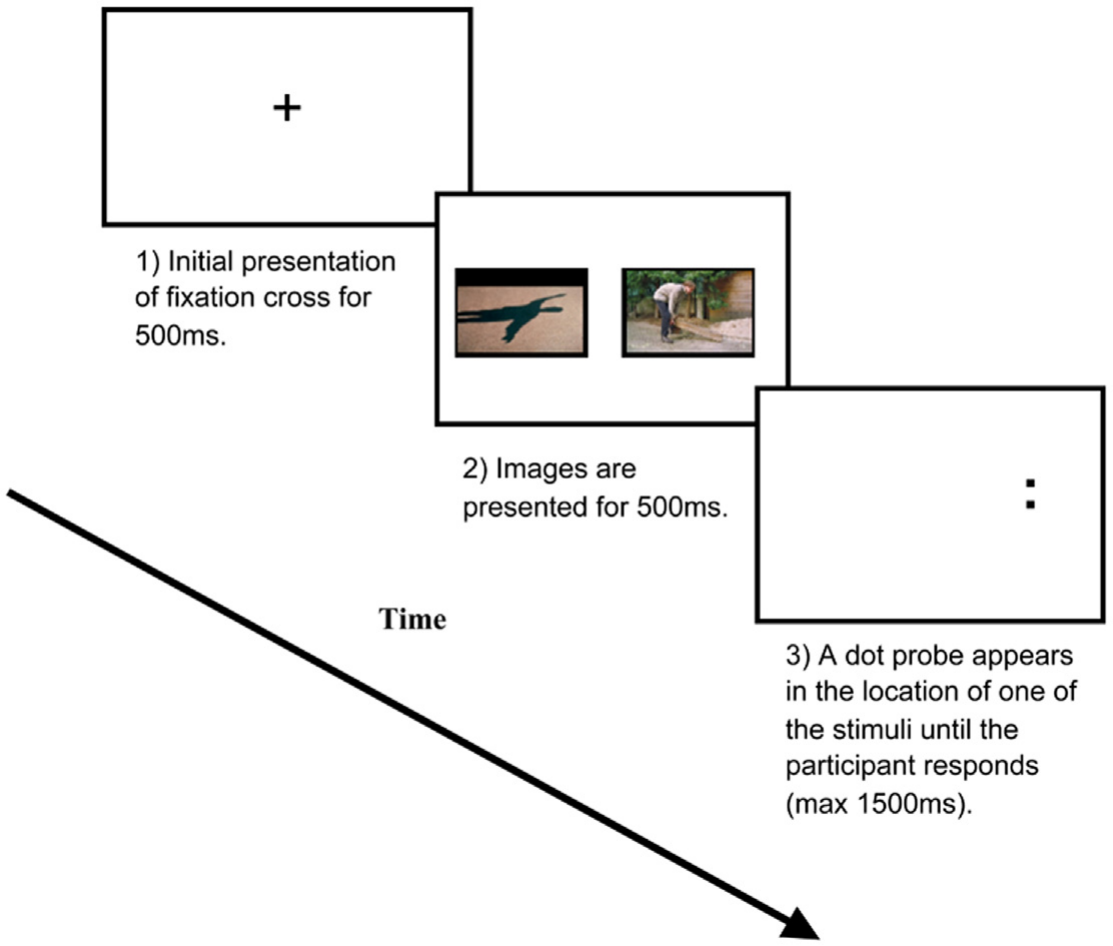
An example of the three stages of the dot probe task in the neutral threat condition.

Participants completed a dot probe task comprising 20 practice trials and 250 experimental trials. The experimental trials were broken down into three blocks of: (i) 100 threat-neutral images; (ii) 100 positive-neutral images; and (iii) 50 neutral-neutral image pairings. The threat images were taken from the Photograph Series of Daily Activities (PHODA) image bank [25] and were back pain specific showing movements known to be associated with evoking pain or pain-related fear (e.g. lifting or bending tasks). These images show everyday activities, which would only represent a pain related threat to those with back pain rather than images of individuals in pain or more generally pain evoking images known to induce a response even in healthy individuals [26]. Although these images have not been rated for valence and arousal, previous research has used the images within dot probe studies [27] and to rate patients’ fear of movement [28]. The positive and neutral images were taken from the International Affective Picture System (IAPS) [29] based on their valence (neutral = 4.5–5.5; positive = 7.3–9.0 (± 1SD)) and arousal ratings (neutral = 4.5–5.5; positive = 1.0–4.1 (± 1SD)) (Image numbers can be found in S1 Appendix). The presentation sequence of the three blocks and the images within each of the blocks were randomized for each participant. Each trial began with a central fixation cross presented for 500ms, followed by an image pair, either, threat-neutral, positive-neutral or neutral-neutral pairs presented for 500ms. The size of each images was 12 x 20 cm, the distance between their inner edges was 9 cm. Images were presented to the left and right of the central point. Following presentation of the image pair, a probe stimulus (a pair of dots aligned either vertically or horizontally) (diameter 7mm) was presented in the location of either the emotional or the neutral image and remained displayed until the participant responded (Fig 1). Participants were asked to press as quickly and as accurately as possible one of two keys on a response button box (the right index finger for [:] and the left index finger for [..]) to identify the probe presented. The inter-trial interval varied randomly between 500 and 1250ms.

### Self-report measures

#### Marlowe-Crowne Social Desirability Scale (MC-SDS-short form) [30]

To assess defensiveness and discriminate defensive high-anxious from high-anxious individuals the 10-item short form of the original MC-SDS (Crowne & Marlowe, 1960) was used. The scale consists of items that are culturally approved but unlikely to occur. For example, “I am always willing to admit it when I make a mistake”. The participants answered either true or false to each statement. Reynolds [31] reported an internal consistency alpha coefficient of 0.66 and a correlation coefficient of r = 0.9 (p< 0.001) was reported between the 10 item MC-SDS and the original 33 item MC-SDS [32], providing support for the shorter version.

#### The State Trait Anxiety Inventory (STAI) [33]

The STAI was used to assess trait anxiety. The scale consists of 20 statements (e.g. “I lack self-confidence”) that participants rate on a scale of 1 (not at all) to 4 (very much so), with a score range of between 20 to 80. The trait component of the STAI has a test-retest reliability of between 0.73 and 0.86 (Spielberger et al., 1970). Eysenck’s theory predicts that cognitive biases will be more evident when state anxiety is high. Since, participants were not asked to rate the perceived threat of the PHODA images, the state component of the STAI questionnaire was administered after the dot probe test to provide a manipulation check to indicate the level of state anxiety. In line with predictions, defensive high-anxious and high-anxious participants scored significantly higher on the state component of STAI compared to the repressor group.

### Procedure

Participants were asked to sit at a desk in a black booth facing a 23inch screen (HP EliteDisplay E231) positioned approximately 70cm in front of them and at eye level, giving a visual angle of 11° between the central fixation cross and the center of the stimulus. A desk mounted chin rest was used to reduce participant head movements, ensuring that the participant’s eyes were level with the middle of the monitor and where the stimuli were presented. Participants were asked to attend to the fixation cross before each trial to standardize the starting location of their gaze. Trials were presented in three blocks to allow participants to rise and move around to accommodate any discomfort experienced by prolonged sitting. After completing the dot-probe task, participants completed the various self-report questionnaires described above.

### Data analysis

#### Dot probe preparation

Attentional bias scores for threatening images relative to neutral were calculated for each participant from the reaction time data of the dot-probe trials using the formula:

((Trpr + Tlpl)/2)−((Nrpr + Nrpl + Nlpr + Nlpl)/4)

Where T = threat, N = neutral, p = probe, r = right position, l = left position.

The attentional bias scores for positive images relative to neutral were calculated for each participant from the reaction time data with the formula:

((Prpr + Plpl)/2)−((Nrpr + Nrpl + Nlpr + Nlpl)/4)

Where P = positive, N = neutral, p = probe, r = right position, l = left position.

Therefore, positive reaction times indicate faster reactions times, therefore attention to emotional image and negative values reflect avoidance of the emotional image. The biases were calculated using this method, rather than comparing congruent to incongruent trials [19] because of differences that have previously been shown in the attentional biases of the four groups affecting the difference between congruent and incongruent trials [18]. Therefore, this method provides a more stable baseline (neutral/neutral reactions time) from which to compare the valenced (threat/positive) trials and the potential to separately consider the effects of congruent and incongruent trials. Reaction times shorter than 200ms or longer than 1200ms were removed from the data. Incorrect responses were also excluded from the analysis. Data from one individual was excluded from the study since their attentional bias score for both threat and positive images was more than two standard deviations from the mean. Errors and outliers accounted for 2.5% of the data.

#### Data analysis overview

Kolmogorov-Smirnov and Levenes’ test indication that the data was normally distributed and the variances were equal, therefore parametric tests could be run. A Multivariate Analysis of Variance (MANOVA) was performed on the attentional bias scores, with personality group as the independent variable, and attentional bias to positive and threatening images as dependent variables. A follow-up, between-group Analysis of Variance (ANOVA) was used to identify any differences in attentional bias between the personality groups. To confirm the existence of an attentional bias, t-tests were performed to ensure there was a significant difference from 0 in the mean attentional bias score. T-tests were performed between the control and non-extreme group to identify differences in attentional bias.

## Results

### Trait anxiety and defensiveness: heterogeneity check

A heterogeneity check was performed on the personality groups prior to the main data analysis. The groups were significantly different in both anxiety (*F*(3, 62) = 27.715, p< 0.01) and defensiveness (*F*(3, 62) = 53.015, p< 0.01).

### Comparisons between the non-extreme patients and control group

Attentional bias in the control group was assessed in order to determine whether the PHODA images contained any emotionally relevant content other than that associated with back pain related movements (Table 2). T-tests demonstrated that the control groups attentional bias score to the PHODA images did not differ from 0 (t(19) = 1.04 p > 0.05), indicating that these images were not perceived as threatening by asymptomatic individuals.

**Table 2:** Mean RTs of congruent trials (in ms; standard deviations in brackets) for each condition in the dot probe task for patients with chronic low back pain and asymptomatic controls.

	Defensive high-anxious	High-anxious	Repressors	Non-extreme	Asymptomatic controls
Threat (ms)	549.40 (64.76)	584.52 (56.92)	616.45 (55.80)	591.24 (70.60)	559.37 (54.98)
Positive (ms)	576.78 (69.56)	589.53 (59.91)	568.00 (46.78)	577.48 (73.67)	548.32 (49.65)
Neutral (ms)	577.44 (67.47)	578.01 (50.35)	597.90 (54.56)	586.65 (66.33)	551.34 (55.39)

To determine whether there was any systematic difference in attention towards or away from the PHODA images in the patient population, the attentional biases of the non-extreme patient group and control population were compared. This demonstrated that there was no difference between groups (t(46) = 0.528, p > 0.05). Indicating that, any bias shown in the more extreme personality groups is indicative of their attention towards or avoidance of images perceived as specifically threatening to low back pain.

### Patient group attentional bias scores

The mean reaction time scores for threatening and positive images for the defensive high-anxious, high-anxious, repressor and non-extreme groups are shown in Table 2.

**Fig 2:**
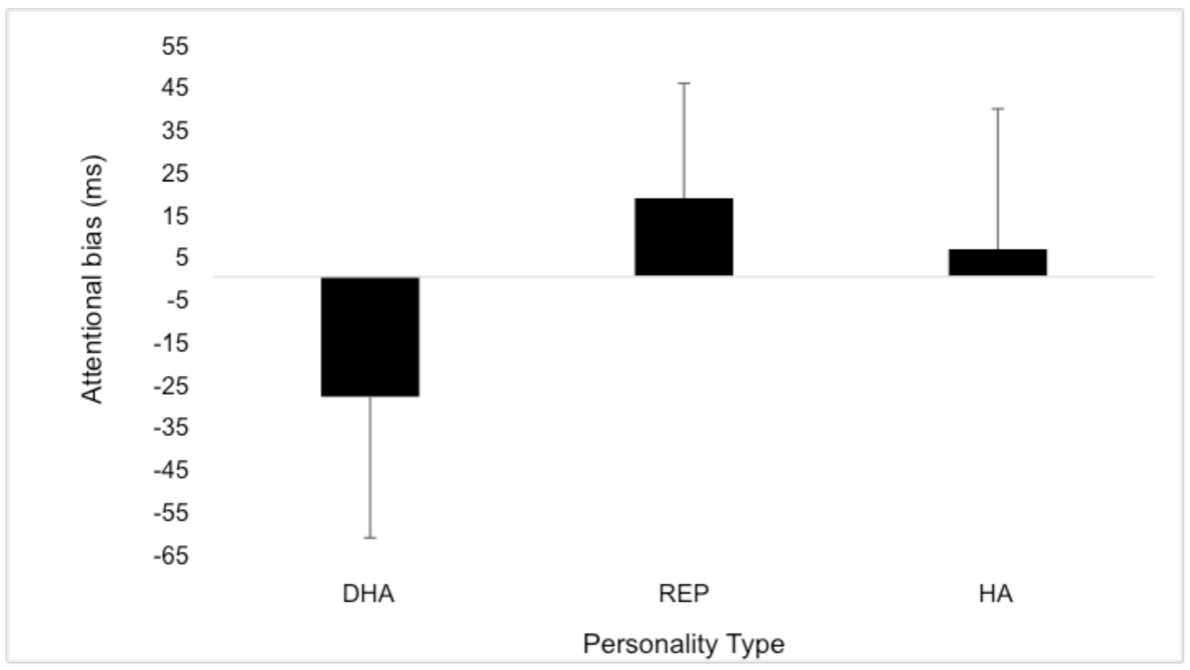
Attentional bias scores (in ms) for threat images for the four groups: defensive high-anxious (DHA), repressor (REP), high-anxious (HA) and non-extreme (NE) groups.

The MANOVA revealed significant between group differences in attentional bias, (Wilks’ Lambda = 0.621, *F*(6, 122) = 5.459, p< 0.01). A follow-up between group ANOVA showed a significant difference in attentional bias for threatening stimuli between personality groups (*F* (3, 62) = 4.573, p< 0.05). *Post hoc* Tukey HSD analysis showed the defensive high-anxious (M = -28.24; SD = ± 33.30) group differed significantly from both the high-anxious (M = 6.51; SD = ± 33.01; effect size = 0.6) and repressor (M = 18.55; SD = ± 26.30 effect size = 0.4) individuals (Fig 2). t-tests showed that the defensive high-anxious group had a significant attentional bias for threatening images compared to neutral (t(17) = 3.59, p < 0.01; effect size = 0.8) whereas the repressors demonstrated a significant avoidant bias away from threatening images relative to neutral (t(8) = 2.11, p < 0.05; effect size = 0.6). The high-anxious and non-extreme individuals showed no bias.

**Fig 3:**
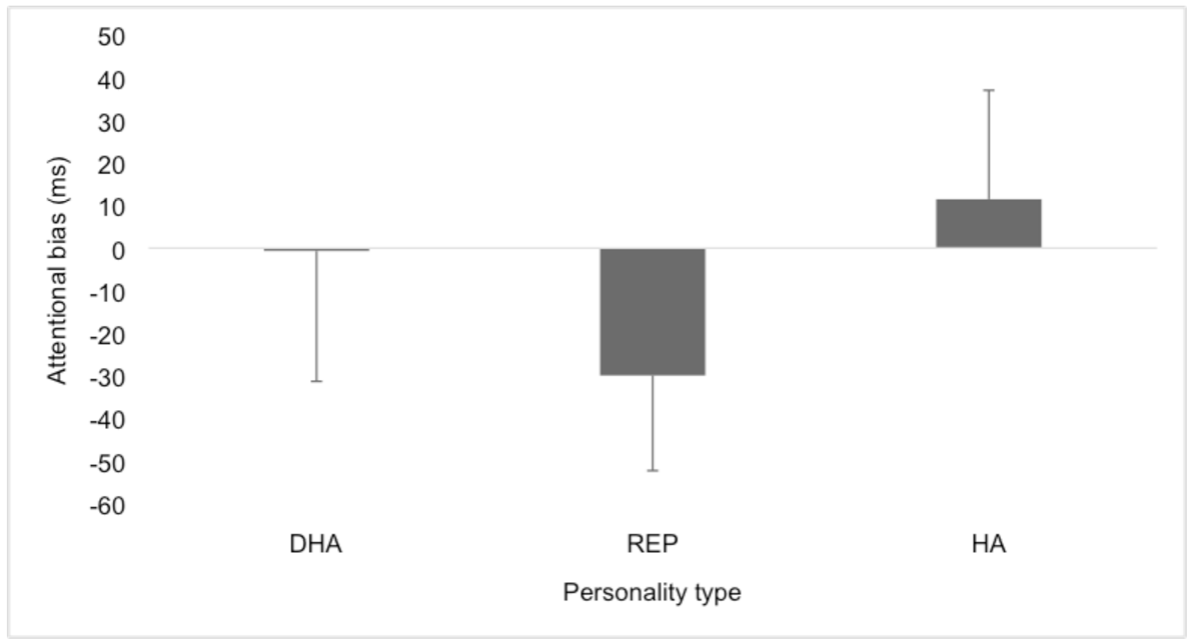
Attentional bias scores (in ms) for positive images for the four groups: defensive high-anxious (DHA), repressor (REP), high-anxious (HA) and non-extreme (NE) groups.

The second ANOVA showed a significant difference between groups in attentional bias for positive stimuli (*F* (3, 62) = 2.863, p< 0.05). *Post hoc* Tukey HSD analysis demonstrated the repressor (M = -29.90; SD = ± 22.42) group to be significantly different from both the high-anxious (M = 11.52; SD = ± 25.54; effect size = 0.7) and the defensive high-anxious (M = -0.67; SD = ± 30.61; effect size = 0.5) individuals (Fig 3). T-test analysis demonstrated that the repressor group had a significant attentional bias towards positive compared to neutral images (t(8) = 4.00, p < 0.01; effect size = 0.8), while high-anxious individuals were shown to be avoidant of positive compared to neutral images (t(10) = 1.51, p < 0.05; effect size = 0.4). The defensive high-anxious individuals showed no bias for positive or neutral images.

## Discussion

To our knowledge, this is the first study to investigate the attentional biases demonstrated by Weinberger et al.’s [13] personality types within a chronic musculoskeletal pain population. The study aimed to test and extend Eysenck’s [14] theory of attentional biases in defensive high-anxious individuals when presented with pain-related stimuli. The results of this study offer support for some elements of Eysenck’s [14] predictions with regard to the attentional biases exhibited by the different personality groups. Contrary to Eysenck’s theory, however, there were differences between defensive high-anxious and high-anxious individuals. The defensive high-anxious group were shown to demonstrate attention towards threatening information but showed no bias with respect to positive images, in contrast, high-anxious individuals were avoidant of positive images but demonstrated no bias towards the threatening information. Repressors were avoidant of threatening information and attended selectively to positive stimuli.

It could be suggested, that the combination of high anxiety and defensiveness has an amplifying effect on the perception of threat resulting in the greater attentional response in this personality group. Eysenck [14] suggested that defensive high-anxious individuals would be likely to demonstrate similar attentional biases to high-anxious individuals. This study, however, suggests that their biases are different when pain-related threat specific images are used. These findings have important implications for the future assessment of high-anxious and defensive high-anxious individuals who present for pain-related treatments.

Defensive high-anxious individuals are often not included as a separate group in research studies, defensiveness is either not measured or combined with high-anxious individuals to form a single group [18,20,21,34]. This approach may produce contradictory findings between studies as the proportion of defensive high-anxious and high-anxious individuals varies considerably between sample populations. Given the particularly high proportion of defensive high-anxious individuals found within clinical pain populations, it is important that the behaviors of these individuals are investigated further to provide additional understanding of their attentional biases to clinically related threatening information. It has been suggested [19] that defensiveness may affect the attentional resource allocation in response to threatening information differently in those with either high or low trait anxiety. A central tenant of Eysenck’s theory is that high levels of trait anxiety cause an enhanced vigilance towards threatening information and that defensive high-anxious individuals attempt to utilize a defensive strategy, which is ineffective, and leads to elevated levels of anxiety. The enhanced attention to pain-related information in the defensive high-anxious individuals may explain why this sub-group of the population have been found to be more persistent in seeking treatment for their pain [24]. Todd et al.’s [35] review of attentional biases within chronic pain patients suggested that enhanced vigilance to pain related stimuli may cause patients to avoid activities they perceive as painful. Research has suggested that cognitive-behavioral treatments can help to reduce selective attention to pain stimuli [36,37]. The clinical implications of the present findings suggest that interventions within the defensive high-anxious group in particular should focus on educating patients as to how they can control their attention to pain symptoms. Future research within chronic pain populations should investigate interpretive biases and, through the use of eye-gaze technology, identify what patients are focusing on when they view pain images.

The data showed that the high-anxious individuals do not demonstrate a bias towards threatening information. This finding supports Mogg et al. [18] who also found that high-anxious individuals demonstrated no bias when presented with physical threat words. They suggested that this could be due to high-anxious individuals utilizing strategies to counteract their vigilant tendencies. The high-anxious group in this study did show avoidance of positive images as suggested by Eysenck (1997). Eysenck’s four-factor theory suggested that cognitive biases are more evident when state anxiety levels are high. Further research should investigate the attentional biases of chronic back pain patients in specific situations of elevated anxiety. This could be achieved by asking participants to view the images with an intention to imitate the movements observed since this instructional set has been shown to lead to heightened motor evoked potentials in action-observation studies [38].

This study provides further evidence in support of Eysenck’s [14] predictions about repressors within a specific back pain population. Repressors have been suggested to show a vigilance-avoidance pattern towards threatening self-relevant information [39]. Whereby, they demonstrate an initial vigilance and then consciously avoid it, to prevent themselves from experiencing negative affect. Numerous studies have investigated repressor individuals within chronic illness [40], and the general population [12,41]. Previous studies have found a pattern of avoidance of negative information suggesting that the repressor group are consistent in their attentional biases, irrespective of the situation. Due to this avoidance of negative affect, within chronic illness populations (e.g. cancer), patients with a repressive coping style have been suggested to cope more effectively because they are able to intentionally forget more negative emotional material and inhibit their perception of pain related information [42]. Within individuals with chronic musculoskeletal pain, avoidance of pain related information may allow repressors to complete normal activities, thereby reporting lower disability and more effectively self-managing.

There are some limitations within this study, which should be considered. Firstly, the PHODA images used were not assessed for their affective content (valence and arousal). However, these images have been used in previous dot probe studies, and to assess perceived harmfulness of daily activities [27,43]. Furthermore, patients within this study reported that they could attribute the images to their daily life and the activities would be difficult for them to complete at home. Analysis of the control group demonstrated that the PHODA images represented a ‘neutral’ image set for those without back pain, and thus the biases shown in the patient groups can be attributed to the pain specific content of the image set. Secondly, the high-anxious and repressor group were small, however, the effect sizes were moderate to high (0.4–0.8). Future research should recruit larger sample sizes to provide support for these differences. Due to their scarcity in the general population, it was not practical to recruit a control population of defensive high-anxious individuals. However, it is reasonable to assume that these groups would perceive the PHODA images as neutral as well. Finally, the images within this study were not matched for content, contrast and brightness. However, the comparisons within the control group give confidence that any differences in these elements did not distort the findings in any systematic manner.

There are a number of recommendations from this study that will inform future research. First, information about patients’ clinical experiences was not collected in this study; it was assumed that the variance in these factors would be distributed equally between personality groups. Future research should also consider information relating to patients’ clinical history, clinical pain experience and cognitive performance, these data could then be included in the analysis as covariates. The current study presented the images in blocks of the same valence category potentially introducing a possible order effect; research using a more randomized design may be useful.

In summary, the present study provides evidence of an attentional bias to threatening information in the defensive high-anxious group. In addition, the different responses demonstrated by the high-anxious individuals highlight the need to ensure they are investigated as two heterogeneous groups and not conflated to create a single high-anxious population. The present findings contribute to the literature and help to explain the cognitive processes of defensive high-anxious individuals within a musculoskeletal pain population. The demonstration of an attentional bias to pain specific threatening information could explain why defensive high-anxious individuals are more likely to show persistence in the pain management system.

## Supporting Information

S1 Appendix The image numbers of the pictures used for the positive and neutral images are listed in the S1 Appendix. (DOCX)

S1 Table The raw data from this study can be found in the S1 Table. (PDF)
