# Supplementary material for: Personality Type Influences Attentional Bias in Individuals with Chronic Back Pain
Source: PLoS One. 2016 Jan 20;11(1):e0147035. doi: 10.1371/journal.pone.0147035 (PMC4720440; doi:10.1371/journal.pone.0147035)
Supplement: S1 Appendix — (DOCX) [file pone.0147035.s001.docx]

IAPS neutral

1122, 1333, 1350, 1390, 1419, 1450, 1505, 1616, 1645, 1675, 1903, 1908, 1935, 2032, 2038, 2309, 2377, 2446, 2487, 2514, 2518, 2521, 2575, 2580, 2840, 2880, 2980, 3005, 5390, 5395, 5455, 5471, 5500, 5510, 5520, 5531, 5532, 5533, 5535, 5661, 5731, 5740, 5900, 5940, 5950, 6150, 6570.2, 6900, 7000, 7001, 7002, 7003, 7004, 7006, 7009, 7010, 7011, 7012, 7013, 7014, 7016, 7017, 7018, 7019, 7020, 7021, 7025, 7026, 7030, 7031, 7032, 7033, 7034, 7035, 7036, 7037, 7038, 7039, 7040, 7041, 7042, 7043, 7044, 7045, 7046, 7050, 7052, 7053, 7054, 7055, 7056, 7057, 7058, 7059, 7060, 7061, 7062, 7077, 7080, 7081, 7090, 7092, 7095, 7096, 7100, 7130, 7140, 7150, 7160, 7161, 7170, 7175, 7179, 7180, 7182, 7184, 7185, 7187, 7207, 7211, 7217, 7233, 7235, 7236, 7242, 7247, 7255, 7509, 7547, 7590, 7632, 7640, 7700, 7705, 7710, 7820, 7830, 7950, 8211, 8325, 9210, 9360, 9422, 9469.

IAPS positive

1410, 1440, 1441, 1460, 1463, 1500, 1510, 1540, 1590, 1600, 1603, 1604, 1610, 1620, 1630, 1710, 1721, 1722, 1731, 1740, 1750, 1999, 2045, 2058, 2070, 2071, 2260, 5000, 5001, 5010, 5199, 5200, 5201, 5202, 5210, 5220, 5260, 5270, 5300, 5450, 5460, 5470, 5480, 5551, 5594, 5600, 5611, 5623, 5629, 5631, 5660, 5700, 5711, 5725, 5750, 5760, 5764, 5779, 5780, 5781, 5811, 5814, 5820, 5825, 5829, 5830, 5831, 5833, 5836, 5890, 5891, 5910, 5982, 7200, 7220, 7230, 7260, 7270, 7280, 7325, 7330, 7350, 7400, 7405, 7430, 7470, 7480, 7492, 7502, 7545, 7580, 8162, 8163, 8170, 8190, 8501, 8502, 8503, 8510, 8531.
