# Supplementary material for: Personality Type Influences Attentional Bias in Individuals with Chronic Back Pain
Source: PLoS One. 2016 Jan 20;11(1):e0147035. doi: 10.1371/journal.pone.0147035 (PMC4720440; doi:10.1371/journal.pone.0147035)
Supplement: S1 Table — (PDF) [file pone.0147035.s002.pdf]

Participant number	Age	Sex	Marlowe-Crowne	Trait anxiety	State anxiety	Attentional bias threat	Attentional bias positive
ZFDP01	53	1	10	33	34	18.34	89.32
ZFDP02	70	1	5	36	30	54.70	3.30
ZFDP03	24	0	4	53	30	33.16	54.53
ZFDP04	48	1	4	31	39	-20.14	-57.97
ZFDP05	55	0	5	39	37	-62.21	-11.40
ZFDP06	43	1	6	59	39	25.56	33.21
ZFDP07	58	1	8	42	31	-9.59	-44.36
ZFDP08	48	1	8	37	25	72.24	-6.74
ZFDP09	66	1	9	31	34	1.80	-30.92
ZFDP10	67	0	8	60	27	-4.50	2.68
ZFDP11	88	1	9	27	21	41.90	-6.31
ZFDP12	63	0	8	44	35	24.77	-44.08
ZFDP13	66	0	6	45	44	-4.08	17.07
ZFDP14	54	0	8	61	34	-80.02	8.98
ZFDP15	27	1	4	49	36	73.39	42.56
ZFDP16	60	0	9	63	41	89.85	113.09
ZFDP17	56	0	8	49	32	-108.17	-60.91
ZFDP18	47	1	6	52	47	-36.57	-38.78
ZFDP19	64	0	5	34	22	-8.89	-105.22
ZFDP20	44	1	9	29	21	6.02	-53.09
ZFDP21	57	1	8	34	29	62.66	-2.98
ZFDP22	58	0	9	56	30	-31.38	6.93
ZFDP23	55	1	9	54	26	-24.79	-49.39
ZFDP24	59	1	10	66	61	27.72	30.10
ZFDP25	34	0	9	40	33	-6.50	-8.87
ZFDP26	36	0	6	49	31	7.93	32.26
ZFDP27	37	0	6	40	40	-7.72	-31.53
ZFDP28	29	1	10	36	34	4.19	-4.10
ZFDP29	49	1	8	41	24	-39.18	14.94
ZFDP30	69	1	8	49	25	-16.31	22.09
ZFDP31	42	1	7	38	35	-129.04	-89.62
ZFDP32	48	1	10	40	30	-51.88	19.29
ZFDP33	30	1	10	42	34	-18.09	49.83
ZFDP34	64	1	9	39	20	28.14	19.98
ZFDP35	67	1	8	50	36	-43.59	2.19
ZFDP36	45	0	10	20	20	2.65	-2.72
ZFDP37	60	1	7	45	26	0.00	0.00
ZFDP38	41	1	6	43	34	0.00	0.00
ZFDP39	61	1	5	27	20	0.00	0.00
ZFDP40	41	1	7	34	28	0.00	0.00
ZFDP41	61	1	8	30	23	0.00	0.00
ZFDP42	39	1	7	49	50	0.00	0.00
ZFDP43	39	1	5	34	26	0.00	0.00
ZFDP44	62	0	6	29	25	0.00	0.00
ZFDP45	28	1	3	56	29	0.00	0.00
ZFDP46	40	0	9	22	20	0.00	0.00
ZFDP47	58	0	6	27	21	0.00	0.00

ZFDP48	38	1	4	61	25	0.00	0.00
ZFDP49	49	1	2	23	20	0.00	0.00
ZFDP50	28	0	7	37	21	0.00	0.00
ZFDP51	71	1	4	40	29	0.00	0.00
ZFDP52	55	1	5	38	20	0.00	0.00
ZFDP53	70	0	7	35	32	0.00	0.00
ZFDP54	56	1	6	33	26	0.00	0.00
ZFDP55	30	1	8	55	23	0.00	0.00
ZFDP56	65	0	8	29	26	42.97	-34.41
ZFDP57	33	0	2	62	22	-6.56	5.44
ZFDP58	43	1	4	40	40	-10.73	11.49
ZFDP59	31	1	3	58	23	-11.86	2.50
ZFDP60	40	1	6	47	27	-1.57	-38.87
ZFDP61	60	1	3	64	22	-8.29	13.14
ZFDP62	44	1	9	57	23	-50.13	4.18
ZFDP63	35	1	8	59	38	-16.46	33.95
ZFDP64	53	1	4	50	36	44.49	32.81
ZFDP65	57	0	4	31	33	2.24	3.61
ZFDP66	38	1	8	41	29	-48.13	-2.30
ZFDP67	54	1	4	41	33	-18.03	13.71
ZFDP68	69	1	8	25	22	49.11	-12.77
ZFDP69	44	0	8	29	31	27.65	-27.66
ZFDP70	30	1	8	25	20	18.85	-17.54

Mean RT Threat	Mean RT Positive	Neutral RT	Trials excluded	% errors
677.71	748.69	659.36	11	4.4
582.27	530.87	527.57	8	3.2
658.82	680.18	625.66	8	3.2
577.35	539.52	597.49	5	2.0
468.88	519.68	531.09	13	5
593.53	601.17	567.97	5	2.0
524.66	489.89	534.25	6	2.4
609.47	530.50	537.23	11	4.4
619.10	586.38	617.30	12	4.8
482.23	485.83	483.15	12	4.8
572.41	524.20	530.51	6	2.4
687.06	618.21	662.29	10	4
736.72	757.87	740.81	8	3.2
457.26	546.27	537.28	12	4.8
662.73	631.90	589.34	6	2.4
549.32	572.56	461.94	6	2.4
537.00	584.26	645.17	8	3.2
596.27	594.06	632.84	5	2
597.71	501.37	606.60	8	3.2
582.96	523.85	576.93	3	1.2
653.34	587.70	590.68	6	2.4
550.31	588.62	581.69	5	2
546.59	521.99	571.38	10	4
542.27	544.65	514.55	6	2.4
457.62	455.24	464.11	4	1.6
507.13	531.46	499.20	5	2
467.16	443.35	474.88	6	2.4
551.49	543.20	547.30	4	1.6
625.65	679.77	664.83	11	4.4
648.38	686.78	664.69	10	4
478.51	517.93	607.55	8	3.2
612.95	684.12	664.831	11	4.4
515.98	583.91	534.08	5	2
646.21	638.06	618.08	9	3.6
518.05	563.83	561.64	4	1.6
511.46	506.08	508.81	4	1.6
0.00	0.00	591.87	7	2.8
0.00	0.00	462.26	9	3.6
0.00	0.00	592.31	2	0.8
0.00	0.00	466.34	4	1.6
0.00	0.00	676.98	8	3.2
0.00	0.00	533.00	3	1.2
0.00	0.00	639.28	4	1.6
0.00	0.00	578.54	8	3.2
0.00	0.00	561.06	4	1.6
0.00	0.00	576.93	7	2.8
0.00	0.00	647.94	4	1.6

0.00	0.00	533.00	3	1.2
0.00	0.00	505.99	9	3.6
0.00	0.00	581.14	4	1.6
0.00	0.00	676.63	4	1.6
0.00	0.00	662.56	4	1.6
0.00	0.00	636.02	4	1.6
0.00	0.00	657.48	7	2.8
0.00	0.00	534.25	6	2.4
674.06	596.68	631.09	4	1.6
526.44	538.44	533.00	3	1.2
544.16	566.37	554.88	5	2
521.14	535.50	533.00	3	1.2
617.31	580.01	618.88	6	2.4
524.71	546.14	533.00	3	1.2
499.80	554.12	549.94	8	3.2
540.69	591.10	557.16	0	0
619.50	607.82	575.01	6	2.4
553.52	554.89	551.29	2	0.8
620.58	666.41	668.71	4	1.6
625.48	657.23	643.51	3	1.2
682.88	621.00	633.77	4	1.6
634.10	578.79	606.45	5	2
658.47	622.08	639.62	5	2
